# Molecular Prevalence and Phylogenetic Analysis of *Anaplasma* spp. in Goats from Adana, Türkiye

**DOI:** 10.3390/vetsci12050481

**Published:** 2025-05-15

**Authors:** Tülin Güven Gökmen, Armağan Erdem Ütük, Esra Aslan Tokgöz, Nur Sima Uprak, Afra Sena Tekin, Ufuk Erol, Pınar Ayvazoğlu Demir, Osman Sezer, Elçin Günaydın

**Affiliations:** 1 Department of Microbiology, Ceyhan Veterinary Faculty, Çukurova University, Adana 01330, Türkiye; nuprak@cu.edu.tr; 2 Department of Parasitology, Ceyhan Veterinary Faculty, Çukurova University, Adana 01330, Türkiye; autuk@cu.edu.tr (A.E.Ü.); astekin@cu.edu.tr (A.S.T.); 3 Adana Veterinary Control Institute, Adana 01122, Türkiye; easlan@tagem.gov.tr (E.A.T.); osman.sezer@tarimorman.gov.tr (O.S.); 4 Department of Parasitology, Veterinary Faculty, Sivas Cumhuriyet University, Sivas 58140, Türkiye; ufukerol@cumhuriyet.edu.tr; 5 Department of Animal Health Economics and Management, Veterinary Faculty, Kırıkkale University, Kırıkkale 71450, Türkiye; pinardemir@kku.edu.tr; 6 Department of Microbiology, Veterinary Faculty, Kastamonu University, Kastamonu 37150, Türkiye; elcingunaydin@kastamonu.edu.tr

**Keywords:** *A. ovis*, *A. phagocytophilum*-like 1, *A. phagocytophilum*-like 2, *A. capra*, goat

## Abstract

Anaplasmosis is a disease in animals that leads to significant economic losses. In addition, the zoonotic potential of vector-borne *Anaplasma* species is increasing its importance, both around the world and in Türkiye, in particular. The aim of this study was to determine the prevalence and phylogenetic profile of *Anaplasma* species in goats from the districts of Adana province, Türkiye, via molecular methods. For this purpose, blood samples from 364 goats of various breeds, ages, and genders were collected, and the presence of 55 *Anaplasma* spp. was determined through the PCR method. *Anaplasma ovis* was detected in 55 samples (15.1%) and *A. phagocytophilum* in 11 samples (3%) by Nested-PCR. However, *A. capra* was not detected. The phylogenetic relationships of *A. ovis* and *A. phagocytophilum* were investigated by DNA sequencing of the *msp4* and *16S rRNA* gene regions. Through sequence analysis in *A. phagocytophilum*, we identified nine isolates as *A. phagocytophilum*-like 1 and one isolate as *A. phagocytophilum*-like 2. Forty-six of the *A. ovis* isolates were 100% similar to each other. The other 2 isolates were also 100% similar to each other but had a single nucleotide difference from the other 46 isolates. Notably, the isolate identified as *A. phagocytophilum*-like 2 was detected for the first time in goats in Türkiye.

## Simple Summary

Anaplasmosis is a very important disease due to the economic losses it causes in animals and the potential for transmission to humans via ticks. Adana province is a province with a suitable climate and geography for the spread of anaplasmosis. In relation to intensive goat farming, this study aimed to investigate *Anaplasma* agents and related strains in goats. Blood samples from a total of 364 goats were collected, and the presence of 55 *Anaplasma* spp. was assessed by PCR. In this study, *A. ovis* was detected in 55 samples (15.1%) and *A. phagocytophilum* in 11 samples (3%). Nine *A. phagocytophilum* isolates were detected as *A. phagocytophilum*-like 1 and one isolate as *A. phagocytophilum*-like 2 by DNA sequencing. *Anaplasma ovis* isolates were categorized into two groups, with a single nucleotide difference between them. Here, the isolate defined as *A. phagocytophilum*-like 2 was detected in goats for the first time in Türkiye. Adana province may be a potential region for the identification of new variants. More frequent epidemiological studies in this region will be useful in managing the Anaplasmosis threat.

## 1. Introduction

Anaplasmosis is an infectious disease caused by *Anaplasma* species that affects both domestic and wild animals in temperate, tropical, and subtropical regions, and it holds significant economic relevance [1]. *Anaplasma* species can infect various cell types and are classified as intracellular pathogens. Specifically, *Anaplasma phagocytophilum* (*A. phagocytophilum*) infects granulocytes in humans and domestic animals, while *Anaplasma marginale* (*A. marginale*), *Anaplasma centrale* (*A. centrale*), and *Anaplasma ovis* (*A. ovis*) target the erythrocytes of ruminants, and *Anaplasma platys* (*A. platys*) infects canine platelets [2]. The transmission of *Anaplasma* species occurs primarily through ticks from the genera *Ixodes*, *Dermacentor*, *Rhipicephalus*, *Haemaphysalis*, *Hyalomma*, *Ornithodoros*, and *Amblyomma* [3].

As with other domestic animals, goats are exposed to species in the *Anaplasma* genus. *A. phagocytophilum* and *A. ovis* are the most frequently identified pathogens in goats [4]. In addition to these species, *Anaplasma bovis* (*A. bovis*), *Anaplasma platys* (*A. platys*), and *Anaplasma capra* (*A. capra*) have also been detected in goat herds from various parts of the world [5,6,7,8]. While anaplasmosis generally manifests as a mild clinical condition in goats, it can develop into a more severe form due to environmental factors, the presence of additional infectious agents, or a compromised immune system. Infected goats may present with clinical symptoms such as high fever, anemia, leukopenia, neutropenia, thrombocytopenia, anorexia, decreased milk production, abortion, infertility, depression, weakness, and weight loss [1,4].

*Anaplasma phagocytophilum* has a broad host spectrum, is usually transmitted by Ixodes (with some exceptions), and poses a threat to public health as a zoonotic pathogen [5]. Recent years have seen the identification of various variants, such as *A. phagocytophilum*-like 1 and *A. phagocytophilum*-like 2. These variants mainly infect sheep, cattle, and goats and are spread by ticks of various genera [6].

Some species in the *Anaplasma* genus are known to cause severe clinical symptoms in animals and humans [1,9]. Therefore, the effective management of Anaplasmosis is of great importance for both veterinary and public health reasons due to the potential economic-related losses in livestock and the zoonotic nature of some *Anaplasma* species. The effective management of the disease also requires developing treatment and control strategies. Early diagnosis, the determination of endemic areas, and phylogenetic analysis are among the basic components of Anaplasmosis control strategies [10].

Understanding these phylogenetic relationships is important for mapping geographical and ecological distributions and host–species relationships. Adana province is located in a transitional area between two regions of Türkiye with quite different climates, which has a diversity of animals and vectors that can host new variants of many agents [11]. However, data on the population structure and phylogenetic relationships of *Anaplasma* species isolated from goats in this region are currently insufficient. The aim of this study was to investigate the presence of *Anaplasma* species in goats obtained from different parts of Adana using Nested-PCR and to determine the phylogenetic relationships of the variants detected through sequencing analyses.

## 2. Materials and Methods

### 2.1. Collection of Blood Samples from Goats and DNA Extraction

Blood samples were collected from goat herds in 15 districts of Adana between June and September 2017. A total of 364 blood samples were taken into collection tubes with EDTA. The animals were clinically healthy and at least six months old, and the samples were stored at −20 °C until DNA isolation. Genomic DNA was extracted from the blood samples of the goats using QIAamp DNA Blood Kits (Qiagen, Hilden, Germany). Genomic DNA was stored at −20 °C until use.

### 2.2. Detection of Anaplasma spp. by 16S rRNA PCR

The 16S ribosomal RNA (*16S rRNA*) gene region of *Anaplasma/Ehrlichia* species was amplified by EC9 and EC12A “catch-all” primers (Table 1). In a 25 µL final volume, 45 mM Tris–HCl, pH 8.8; 11 mM (NH_4_)_2_SO_4_; 2.5 mM MgCl_2_; 0.8 mM each of dATP, dCTP, dGTP, and dTTP; 1 U Taq DNA polymerase (Solis biodyne, Tartu, Estonia); 10 µM EC9 and EC12A; and 2 µL genomic DNA sample were used. The PCR assays were performed as described previously [12]. The thermal cycling protocol used was as follows: an initial denaturation phase of 5 min at 94 °C, followed by 35 cycles of 94 °C for 1 min, 58 °C for 1 min, 72 °C for 1 min, and, finally, an additional extension phase at 72 °C for 7 min. Amplicons were subjected to 1.5% agar gel electrophoresis with ethidium bromide at 100 V for 60 min, and, after electrophoresis, they were visualized with a gel imaging system. A 50–2000 bp marker was used to determine the band size.

### 2.3. Investigation of A. phagocytophilum by the Nested-PCR Method

The SSAP2F and SSAP2R primers specific to the *16S rRNA* region were used for the detection of *A. phagocytophilum* (Table 1). In the PCR, 45 mM Tris–HCl, pH 8.8; 11 mM (NH_4_)_2_SO_4_; 2.5 mM MgCl_2_; 0.8 mM each of dATP, dCTP, dGTP, and dTTP; 1 U Taq DNA polymerase (Solis biodyne, Tartu, Estonia); 10 µM primers; and 1 µL EC9/EC12A Nested-PCR amplicon were employed in a final volume of 25 µL. The PCR assays were performed as described previously [13]. The thermal cycling protocol used was as follows: an initial denaturation phase of 5 min at 94 °C, followed by 35 cycles of 94 °C for 35 s, 55 °C for 40 s, 72 °C for 40 s, and, finally, an additional extension phase at 72 °C for 10 min. Amplicons were run on a 1.5% agarose gel at 100 V for 1 h and visualized with a UV transilluminator.

### 2.4. Investigation of A. ovis by the msp4 Nested-PCR Method

msp4F and msp4R primers were used to detect the msp4 gene region (Table 1). At a final volume of 25 µL, 45 mM Tris–HCl, pH 8.8; 11 mM (NH_4_)_2_SO_4_; 2.5 mM MgCl_2_; 0.2 mM each of dATP, dCTP, dGTP, and dTTP; 1.25 U Taq DNA polymerase (Solis biodyne, Tartu, Estonia); 0.4 µM primers; and 1 µL *16S rRNA* Nested-PCR amplicon were used. The PCR assays were performed as described previously [14]. The thermal cycling protocol used was as follows: an initial denaturation phase of 10 s at 94 °C, followed by 30 cycles of 94 °C for 30 s, 62 °C for 15 s, 72 °C for 30 s, and, finally, an additional extension phase at 72 °C for 5 min. Amplicons were run on a 1.5% agarose gel at a 100 V current for 1 h and visualized with a UV transilluminator.

### 2.5. Investigation of A. capra by the gltA Nested-PCR Method

The Nested-PCR assays were performed as described before [12]. The *gltA* gene region with OuterF/R and InnerF/R primers is shown in Table 1. In the first step, PCR reactions were performed with a total volume of 25 μL containing 45 mM Tris–HCl, pH 8.8; 11 mM (NH_4_)_2_SO_4_; 2.5 mM MgCl_2_; 0.2 mM each of dATP, dCTP, dGTP, and dTTP; 1.25 U Taq DNA polymerase (Solis biodyne, Tartu, Estonia); 0.4 µM OuterF/R primers; and 1 µL DNA. The PCR conditions comprised initial denaturation for 5 min at 94 °C followed by 30 cycles of denaturation for 45 s at 94 °C, annealing for 45 s at 60 °C, and elongation for 45 s at 72 °C. In the second step of the Nested-PCR, 1 µL OuterF/R amplicon and 0.4 µM InnerF/R primers were added to the master mix. The only difference in amplification conditions was the annealing temperature of 55 °C. Amplicons were run on a 1.5% agarose gel at a 100 V current for 1 h and visualized with a UV transilluminator.

### 2.6. Sequencing and Phylogenetic Analysis

All PCR-positive samples were sequenced. The DNA sequence analyses were performed by a commercial company (Macrogen, Amsterdam, The Netherlands). To perform sequence analysis, the amplicons were purified by use of a clean-up kit (Zymo Research, Irvine, CA, USA). SSAP2F/R and msp4F/R primers were used for the bidirectional sequence analysis. Sequencing was performed using an ABI 3730XL analyzer (Applied Biosystems, Foster City, CA, USA) and a BigDye Terminator v3.1 Cycle sequencing kit (Applied Biosystems, Foster City, CA, USA).

The obtained chromatograms were opened using Geneious Prime 2021.0.3 software (https://www.geneious.com) accessed on 20 September 2024, and their quality scores were examined. A consensus sequence was created by combining the 5′-3′ and 3′-5′ sequences of each isolate using the same program. A local database was created by downloading sequences from the NCBI database (https://www.ncbi.nlm.nih.gov) accessed on 25 September 2024 in order to compare the partial sequences of the *msp4* (*A. ovis*) and *16s rRNA* (*A. phagocytophilum*) genes of *Anaplasma* agents. Phylogenetic trees were created using the reference sequences in the local database with the help of the Mega-11 program [15]. The consensus sequences generated within the scope of this study using the MUSCLE algorithm of the Mega-11 program were aligned and recorded with the nucleotide sequences downloaded from GenBank.

The best models for use in tree creation were determined using the “Find Best Substitution Models” feature of the Mega-11 program. These models were determined as “Kimura-2 + Gamma Distributed with Invariant Sites (G + I)” for *A. phagocytophilum* and “Kimura-2 + Gamma distribution (+G)” for *A. ovis.* Phylogenetic trees were created using the maximum likelihood statistical method in the Mega-11 program. Bootstrap analysis (1000 replications) was performed to determine the reliability of the tree topology.

### 2.7. Statistical Analysis

The data were analyzed using SPSS Statistics 26.0 (IBM Corp., Armonk, NY, USA). Pearson’s Chi-square test was employed to determine the relationships between categorical variables. Prior to analysis, the assumption that at least 80% of the expected frequencies were greater than 5 was checked in order to ensure the validity of the test. A significance level of *p* < 0.05 was adopted for all comparisons.

## 3. Results

**Figure 1:**
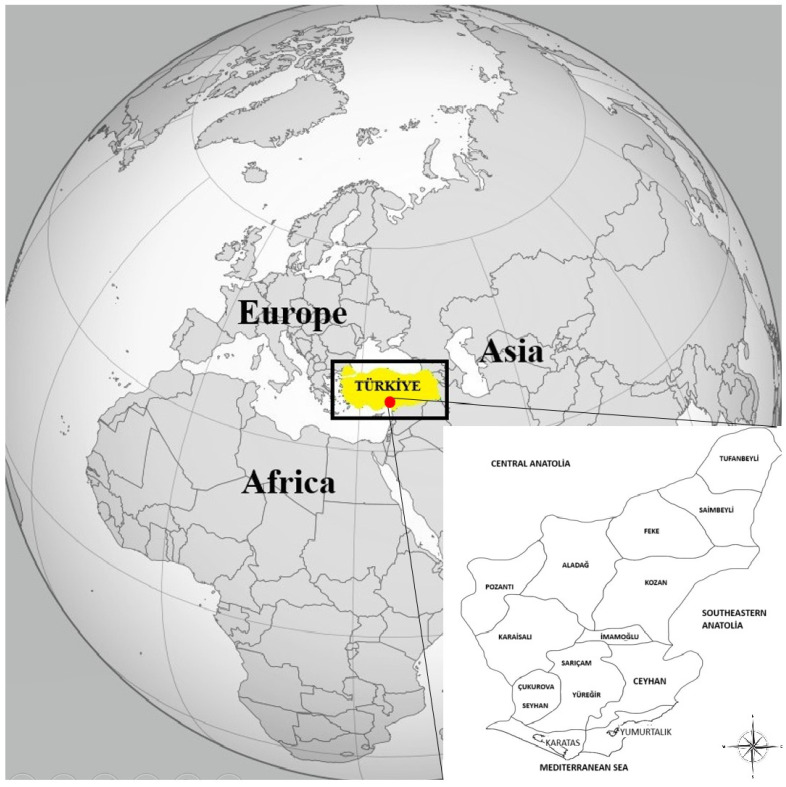
Adana province’s location in the world and in Türkiye. The map was created using QGIS v.3.10.5 software (https://www.qgis.org/en/site/forusers/download.html) accessed on 25 September 2024.

In this study, blood samples of 364 goats were collected from 15 districts of Adana province, showing various geographical and climatic differences (Figure 1).

**Figure 2:**
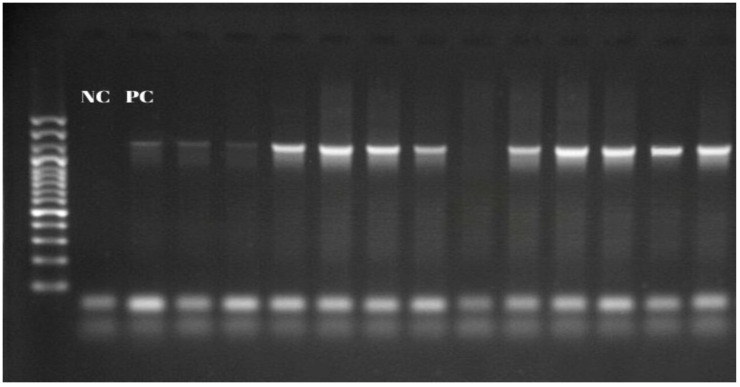
Products of *16S rRNA* PCR.

The presence of *Anaplasma* spp. was investigated by the PCR method with EC9 and EC12A primers and was detected in 55 (15.1%) blood samples. When the distribution of isolates according to districts was examined, the highest prevalence was determined in Tufanbeyli district, at 3.84% (14/364) (Figure 2, Table 2).

The distribution of *Anaplasma* spp. in the goats was also examined according to demographic information, and statistical analysis was performed using the Chi-square test. Of the 55 goats in which *Anaplasma* spp. was detected, 51 (15.5%) were female and 4 (11.1%) were male. According to the Chi-square analysis, the association between gender and the *Anaplasma* spp. rate was not statistically significant (*p* > 0.05). The goats were divided into four age groups (0–1/2–3/4–5/5<). According to the Chi-square analysis, the association between age and *Anaplasma* spp. positivity was not statistically significant (*p* > 0.05) (Table 2).

The goats were examined at the breed level, and the presence of *Anaplasma* spp. was determined at rates of 17.8% in Turkish Hair goats, 17.2% in Aleppo goats, and 3% in other breeds. The associations were found to be statistically significant (*p* < 0.05). In addition, the relationship between the distribution of *Anaplasma* spp. in the goats and the altitude of the districts was investigated. The districts were divided into three groups according to their altitudes (0–50 m, 50–500 m, >500 m). Statistical analysis was performed using the Chi-square test, and the association between altitude and *Anaplasma* prevalence was not found to be statistically significant (*p* > 0.05) (Table 2).

**Figure 3:**
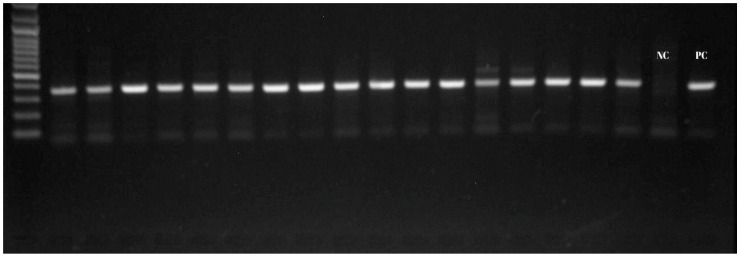
*Anaplasma ovis* msp4 gene Nested-PCR products.

The presence of *A. ovis* was determined by the *msp4* Nested-PCR technique. *A. ovis* was present in all 55 blood samples that had previously tested positive for *Anaplasma* spp. (Figure 3). When the distribution by districts was examined, the highest *A. ovis* prevalence was seen in Tufanbeyli, at 3.84% (Table 2). *Anaplasma capra* was not found in this study.

**Figure 4:**
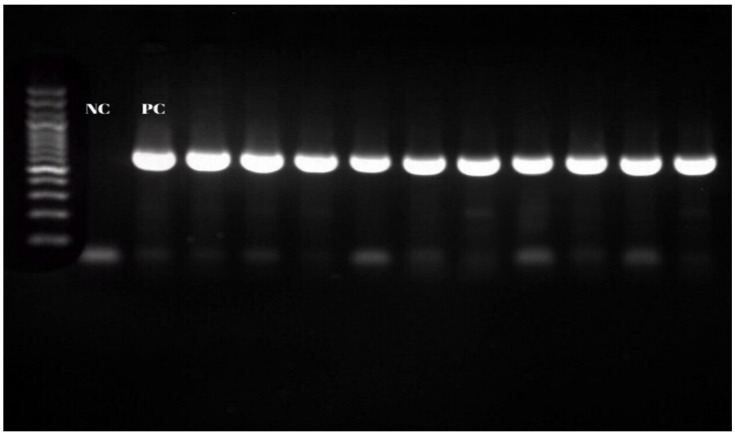
*Anaplasma phagocytophilum 16S rRNA* gene Nested-PCR products.

In the Nested-PCR performed with SSAP2f and SSAP2r primers targeting the *16S rRNA* gene region in the samples positive for *A. phagocytophilum*, 11 (20%) isolates were detected (Figure 4). When the distribution of isolates by districts was examined, the highest prevalence was found in Tufanbeyli, where nine (2.47%—9/364) isolates were detected (Table 2).

In this study, sequence analysis was performed on the *msp4* and *16S rRNA* gene regions for *A. ovis* and *A. phagocytophilum* isolates, respectively, and phylogenetic trees were constructed based on similarities.

**Figure 5:**
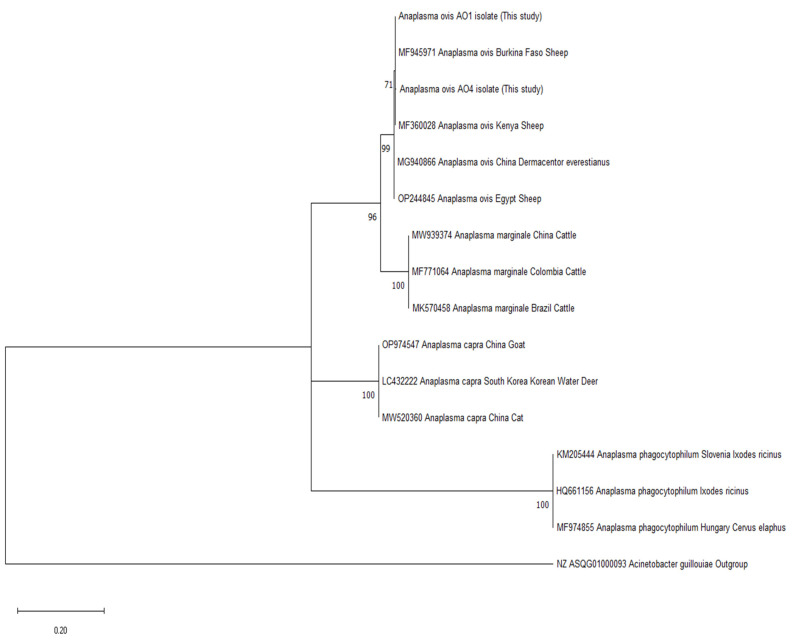
Phylogenetic trees according to the *msp4 gene* sequences of *Anaplasma ovis*, drawn with the maximum likelihood method in Mega-11 software. Numbers at the nodes represent bootstrap values with 1000 replicates. The scale bar shows 0.02 substitutions per nucleotide.

In the *msp4* gene sequence analysis, 48 of the 55 isolates were identified as *A. ovis*, and two variants with single nucleotide differences of 46 and 2 members were detected. In the phylogenetic tree comparing the isolates in this study with the isolates in the genebank, it was determined that the 48 *A. ovis* isolates were in the same clade as sheep isolates from Burkina Faso and Kenya (Figure 5, Table 3).

**Figure 6:**
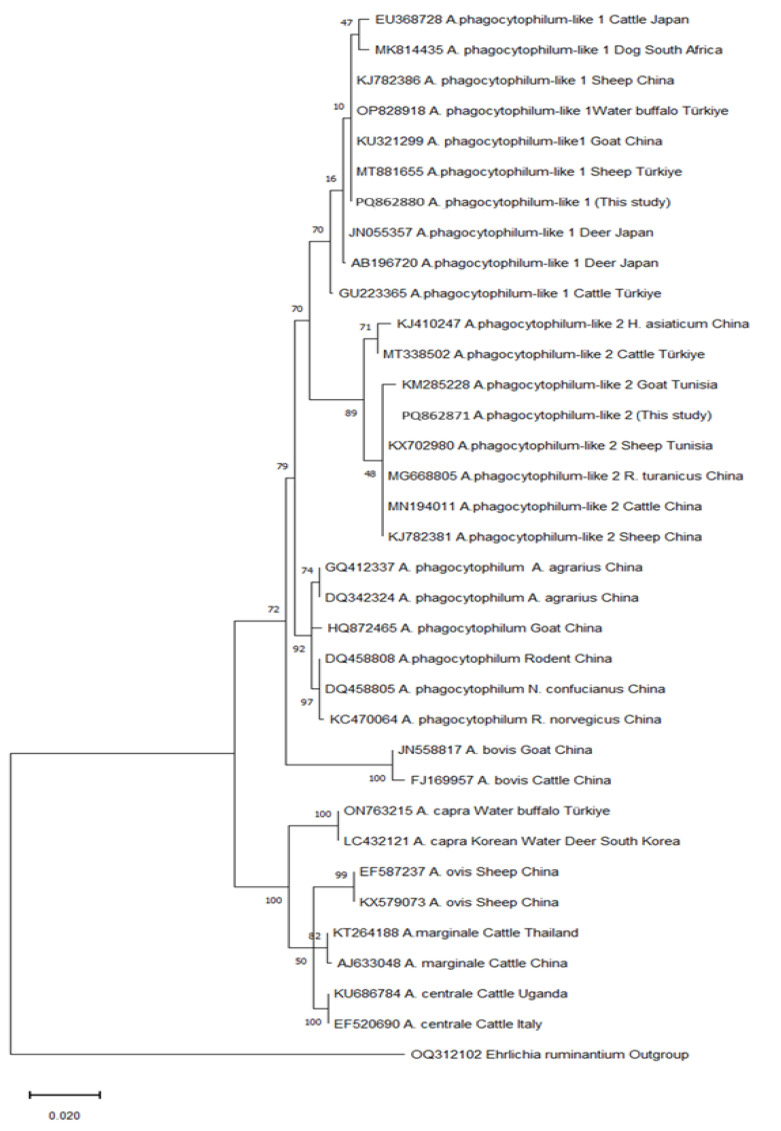
Phylogenetic tree of the *16S rRNA* sequences of *A. phagocytophilum* and related strains, as well as other *Anaplasma* species, drawn with the maximum likelihood method in Mega-11 software. Numbers at the nodes represent the bootstrap values with 1000 replicates. The scale bar shows 0.02 substitutions per nucleotide position.

The *16S rRNA* gene region sequence analysis was performed on 10 *A. phagocytophilum* isolates. Nine isolates were identified as *A. phagocytophilum*-like 1, and one was identified as *A. phagocytophilum*-like 2. In the phylogenetic tree, it was determined that nine *A. phagocytophilum*-like 1 isolates were in the same clade as a buffalo isolate from Türkiye and sheep and goat isolates from China (Table 3). Furthermore, the *A. phagocytophilum*-like 2 isolate was in the same clade as goat and sheep isolates from Tunisia and sheep and cattle isolates from China (Figure 6).

## 4. Discussion

Türkiye, which ranks first among European countries and 23rd in the world in terms of its goat population, is a country where goat breeding is intensively carried out in its Mediterranean, Aegean, and Southeastern Anatolia regions due to the economic value of goat meat and milk [16]. Adana province, where this study was conducted, is among the provinces in Türkiye where the highest numbers of goats are raised [17]. This province borders the Antalya and Mersin provinces, wherein we find 46% of the *Anaplasma* in goats in Türkiye [18].

Goats may encounter more plants and microhabitats compared to sheep and other small ruminants due to their roaming and climbing behaviors. Ticks may cling to goats more easily as their fur is shorter, and the risk of tick infestation may be higher because goats feed on the upper leaves of plants. For this reason, goats are useful for the analysis of Anaplasmosis—a tick-borne disease—in our province. In addition, Adana, which is at the easternmost border of the Mediterranean basin, where the Mediterranean climate transitions into the continental climate, has an altitude of 0–1474 m and is an important location both ecologically and epidemiologically, is a region likely to host new and different variants of *Anaplasma* species in goats.

*Anaplasma ovis* and *A. phagocytophilum* are particularly common in goats and are often unnoticed due to subclinical carriage. However, *Anaplasma* spp. can cause coinfections with hemoparasites, such as *Erlichia*, *Babesia*, and *Theileria*, in goats. These coinfections can increase the severity of the disease and make diagnosis and treatment more difficult. Mixed infections or coinfections put more pressure on the immune system and can ultimately cause a loss of productivity and increased mortality rates [19]. In anaplasmosis, the vector is as important as the presence of *Anaplasma* spp., as it is a tick-borne disease. It is usually carried by ixodidae ticks. In Türkiye, *A. ovis* is mostly carried by *Boophilus*, *Dermacentor*, *Rhipicephalus*, *Hyalomma*, *Ixodes*, and *Ornithodorus* species, while *A. phagocytophilum* has been detected in Ixodes. Adana province has a subtropical climate, forests, and pastures. For this reason, it is a suitable location representing the habitat and seasonal activities of ticks [20]. *Anaplasma phagocytophilum* causes a disease in humans commonly known as human granulocytic anaplasmosis (HGA), which is particularly notable in immunocompromised individuals. HGA can range from an asymptomatic or self-limited infection to a life-threatening infection with multiorgan involvement. Common symptoms include fever, gastrointestinal complaints (most commonly diarrhea), headaches, myalgia, and arthralgia. However, it can also present more seriously with pneumonia, sepsis, myocarditis, peripheral neuropathy, splenic rupture, trigeminal neuralgia, orchitis, cerebral infarction, dysarthria, and hemophagocytic lymphohistiocytosis (HLH) [21]. Following assessments of the agent, vector, and host characteristics, determining the transmission dynamics of the agent, as well as the geographical distribution, prevalence, and phylogenetic relationships of the vector and the agent, is of critical importance for the implementation of effective control strategies, and this requires regular epidemiological studies [22]. Studies on *Anaplasma* species in goats from this region are limited. Therefore, in order to provide epidemiological data from our region and contribute to control strategies, blood samples were collected from 364 goats from 15 districts of Adana province, and the presence of *A. ovis*, *A. phagocytophilum*, and *A. capra* species was investigated.

Among all blood samples, 55 (15.1%) *Anaplasma* spp. isolates were detected by PCR with primers specific to the *Anaplasma/Ehrlichia* spp. *16S rRNA* gene region. The distribution of *Anaplasma* spp. in goats was examined according to breed, age, sex, and the altitude of the districts, and statistical analysis was performed with a Chi-square test. According to the Chi-square analysis, the differences between the rates of *Anaplasma* spp. in goats according to sex and age were not statistically significant (*p* > 0.05). However, when goats were examined at the breed level, it was determined statistically that *Anaplasma* spp. was more prevalent in Turkish Hair goats (*p* < 0.05). In addition, the relationship between the distribution of *Anaplasma* spp. in goats and the altitude of the districts was investigated, but it was not found to be statistically significant (*p* > 0.05). Interestingly, the highest number of goats from which *Anaplasma* was isolated was seen in Tufanbeyli district, and *A. phagocytophilum*-like 2 isolates were also detected there. Additionally, 9 (81.8%) of the 11 *A. phagocytophilum* isolates were detected in Tufanbeyli. Although no relationship was found in terms of altitude, it was estimated that the reason for this higher rate in Tufanbeyli could be related to animal management systems, climate, and the presence and distribution of tick species. The fact that Tufanbeyli district is located on the border of the Central Anatolia Region, where the tick population and diversity are higher, and that its climate is continental, unlike in other Adana districts with a Mediterranean climate, represents important differences (Figure 1). In addition, when investigating satellite maps, it was thought that northern Adana could represent an ecological area where tick populations are dense, as it is a district located on the forest–steppe border.

When *Anaplasma* isolates were investigated at the species level, the presence of *A. ovis* (100%) was determined in all 55 *Anaplasma* spp. isolates with a primer specific to the *msp4* gene region. The prevalence of *A. ovis* was 15.1% (55/364). In countries bordering the Mediterranean, the prevalence rates in goats were 33% in Egypt, 65–70% in Tunisia, and 54% in Algeria [23,24,25,26]. In the neighboring countries of Iran and Iraq, the prevalence in goats was 54% and 66.6%, respectively [27,28]. In Türkiye, *A. ovis* prevalence was determined as 15–50% in various studies [18,29,30,31,32]. In a study conducted in Adana in 2018, *A. ovis* was found in 7 (18.9%) of 37 goat blood samples, which is consistent with this study’s results. However, the most comprehensive sampling in Adana was performed in this study [30].

Of the 55 isolates identified as *A. ovis*, 48 were identified by *msp4* gene sequence analysis, and two variants of *A. ovis*, one containing 46 isolates and the other containing 2 isolates, were identified. Only a single nucleotide was detected to be different between them. This mutation was a silent mutation as it did not cause any change in the amino acid sequence of major surface protein 4 (Serine: TCT→TCC) (Table 3). In addition, the presence of only a single nucleotide mutation indicates low genetic diversity in this region. There are limited evolutionary differences between the strains. As such, Adana is a genetically homogeneous region for *A. ovis*. It was observed that the two variants were in the same clade in the phylogenetic tree as sheep and goat isolates identified in China, Egypt, Kenya, and Burkina Faso (Figure 5). This clade probably spread from an ancestral strain of Asian–African origin, and its genetic differentiation may be slow.

With *16S rRNA* gene-region-specific primers, *A. phagocytophilum* was detected in 11 out of 55 *Anaplasma* spp., and the prevalence was determined as 3% (11/364). This result shows that 11 goats had *A. ovis/A. phagocytophilum* coinfection. In Türkiye, *A. phagocytophilum* prevalence was determined as 28.04% in the Mediterranean region, 5.8% in Thrace, 1.5% in Şanlıurfa, and 43.18% in Sivas [6,33,34,35]. When countries were examined, the prevalence was found to be 1.6–2.5% in Tunisia, 33% in Saudi Arabia, and 23% in China. In Switzerland, the prevalence was 5.6% [13,24,36,37]. However, it has been reported that the prevalence is higher in some countries; for example, PCR positivity was found in four out of five goats in Northern Ireland. In North America, this rate varies between 6.3% and 38.2% [38,39]. The prevalence we detected in this study is similar to that shown in other data from Türkiye, Italy, and Tunisia.

In this study, the *16S rRNA* gene regions of 10 of the *A. phagocytophilum* isolates were sequenced and identified as *A. phagocytophilum*-related strains, including *A. phagocytophilum*-like 1 and *A. phagocytophilum*-like 2 isolates. *Anaplasma phagocytophilum*-like 1 is a variant detected in large and small ruminants and dogs and is seen in China, Japan, Thailand, Kyrgyzstan, Tunisia, and Türkiye. In previous studies in Türkiye, it was detected in sheep, goats, buffalos, cattle, and ticks in the provinces of Sivas, Malatya, Bingöl, Elazığ, Muş, Mersin, and Antalya [6,40,41,42,43,44,45,46]. In the phylogenetic tree, it was determined that nine *A. phagocytophilum*-like 1 isolates with 100% similar DNA profiles had bootstrap values close to those of sheep, goat, and buffalo isolates detected in studies conducted in Türkiye and of sheep and goat isolates detected in China, with which they were in the same clade (Figure 5, Table 3).

*Anaplasma phagocytophilum*-like 2 variants have been isolated in cattle, sheep, and goats in Senegal, Tunisia, and China [5,40,47]. In Türkiye, *Anaplasma phagocytophilum*-like 2 was previously detected together with *Anaplasma phagocytophilum*-like 1 in sheep and cattle and in various ticks. It was also searched for in buffalo but could not be found [6,43]. In this study, *Anaplasma phagocytophilum*-like 2 variants were detected in goats for the first time in Türkiye. Studies on *A. phagocytophilum*-related strains in Türkiye started in 2021. These studies were conducted on ruminants and ticks. As far as we can see, *A. phagocytophilum*-like 1 was only assessed in two studies conducted on goats, and *A. phagocytophilum*-like 2 was not found [6,33]. In the phylogenetic tree, the *A. phagocytophilum*-like 2 isolate had a bootstrap value close to those of sheep, goat, cattle, and tick isolates detected in Tunisia and China, with which it was in the same clade (Figure 6).

*Anaplasma phagocytophilum*-like 1 and 2 variants have been detected in various parts of the world, such as Türkiye, Kyrgyzstan, Korea, China, Senegal, and Tunisia [5,6,7,13,24,33,44,45,46,47]. However, the detection of *A. phagocytophilum*-related strains in certain regions is probably linked to the studies being conducted in these regions. If studies are conducted in different places, it is likely that these variants will also be detected in other parts of the world. The clade and bootstrap values obtained in the phylogenetic trees constructed with our isolates and isolates from other countries are quite close to each other, and, therefore, no significant epidemiological differentiation could be made. However, although the pathogenicity, symptoms, potential vectors, and zoonotic potential of these variants are not clearly known, they need to be closely monitored due to their close phylogenetic relationship with *A. phagocytophilum*, which has a wide host range and pathogenic properties.

## 5. Conclusions

This study presented detailed demographic data on the distribution and prevalence of *A. ovis*, *A. phagocytophilum*-like 1, and *A. phagocytophilum*-like 2 in goats in Adana—an important province in Türkiye for goat breeding—focusing on 15 districts. *A. phagocytophilum*-like 2 was reported in goats for the first time in Türkiye in this study. The detection of *A. phagocytophilum*-like 2 in goats in our region, and the fact that the region assessed is transitional in terms of its climate and vegetation, together necessitate further studies on the species distribution of ticks.

## Figures and Tables

**Table 1:** Primers used for amplification of the EC9/12A *16S rRNA*, *msp4*, and *gltA* genes.

Primer	Sequences	bp	Genus/Species
EC9 EC12A	5′-TACCTTGTTACGACTT-3′ 5′-TGATCCTGGCTCAGAACGAACG-3′	1462 bp	*Anaplasma/Ehrlichia*
SSAP2F SSAP2R	5′-GCTGAATGTGGGGATAATTTAT-3′ 5′-ATGGCTGCTTCCTTTCGGTTA-3′	641 bp	*Anaplasma phagocytophilum*
msp4F msp4R	5′-TGAAGGGAGCGGGGTCATGGG-3′ 5′-GAGTAATTGCAGCCAGGCACTCT-3′	347bp	*Anaplasma ovis*
OuterF OuterR InnerF InnerR	5′-GCGATTTTAGAGTGYGGAGATTG-3′ 5′-TACAATACCGGAGTAAAAGTCAA-3′ 5′-TCATCTCCTGTTGCACGGTGCCC-3′ 5′-CTCTGAATGAACATGCCCACCCT-3′	1031 bp 594 bp	*Anaplasma capra*

**Table 2:** Distribution of *Anaplasma* spp. in goats according to age, breed, gender, altitude, and districts.

**Age** X2 = 1.969 *p* = 0.579 *p* > 0.05	**Positive**	**Negative**	**Total**
≤1	6 (10.9%)	49 (89.1%)	55 (100.0%)
2–3	12 (16.2%)	62 (83.8%)	74 (100.0%)
4–5	18 (18.75%)	78 (81.25%)	96 (100.0%)
>5	19 (13.6%)	120 (86.4%)	139 (100.0%)
**Gender** X2 = 0.563 *p* = 0.453 *p* > 0.05			
Female	51(15.5%)	277 (84.5%)	328 (100.0%)
Male	4 (11.1%)	32 (88.9%)	36 (100.0%)
**Breed** X2 = 9.054 *p* = 0.011 *p* < 0.05			
Turkish Hair goat	43 (17.8%)	198 (82.2%)	241 (100.0%)
Aleppo goat	10 (17.2%)	48 (82.8%)	58 (100.0%)
Others	2 (3%)	63 (97%)	65 (100.0%)
**Altitude** X2 = 3.741 *p* = 0.154 *p* > 0.05			
0–50 m	18 (15.1%)	101 (84.9%)	119 (100.0%)
50–500 m	13 (10.65%)	109 (89.35%)	122 (100.0%)
>500 m	24 (19.5%)	99 (80.5%)	123 (100.0%)
Districts			
Ceyhan	6 (20%) ***A. ovis* (6)**	24 (80.0%)	30 (100.0%)
Yumurtalık	5 (20.8%) ***A. ovis* (5)**	19 (79.2%)	24 (100.0%)
Kozan	1 (4.2%) ***A. ovis* (1)**	23 (95.8%)	24 (100.0%)
İmamoğlu	6 (25%) ***A. ovis* (6)**	18 (75%)	24 (100.0%)
Sarıçam	2 (7.4%) ***A. ovis* (2)**	25 (92.6%)	27 (100.0%)
Karataş	-	12 (100.0%)	12 (100.0%)
Yüreğir	1 (4%) ***A. ovis* (1)**	24 (96%)	25 (100.0%)
Çukurova	-	24 (100.0%)	24 (100.0%)
Karaisalı	4 (16.6%) ***A. ovis* (4)**	20 (83.4%)	24 (100.0%)
Saimbeyli	1 (4%) ***A. ovis* (1)**	24 (96%)	25 (100.0%)
Tufanbeyli	14 (58.3%) ***A. phagocytophilum* (9)** ***A. ovis* (14)**	10 (41.7%)	24 (100.0%)
Feke	-	24 (100.0%)	24 (100.0%)
Pozantı	3 (11.5%) ***A. phagocytophilum* (1)** ***A. ovis* (3)**	23 (88.5%)	26 (100.0%)
Aladağ	6 (24%) ***A. ovis* (6)**	19 (76%)	25 (100.0%)
Seyhan	6 (23%) ***A. phagocytophilum* (1)** ***A. ovis* (6)**	20 (77%)	26 (100.0%)
Total	55 (15.1%)	309 (84.9%)	364 (100.0%)

**Table 3:** *Anaplasma* spp. GenBank number and nucleotide substitution localization.

Genus/Species/N		GenBank Numbers	Nucleotide Substitution Localization																
*Anaplasma* spp. PCR-Positive 55 (15%)	*A. ovis* 55 (100%)	*A. ovis genotype 1* (46 isolates) PQ878024–PQ878036 PQ878039–PQ878071 *A. ovis genotype 2* (2 isolates) PQ878037, PQ878038	GenBank Accession Numbers	Country	Host	*msp4* Nucleotide Positions													
						188													
			PQ878024 **	TR	Goat	T													
			PQ878037 **	TR	Goat	C													
			MF945971	BF	Sheep	T													
			MF360028	KE	Sheep	T													
	*A. phagocytophilum* 11 (20%)	*A. phagocytophilum*-like 1 PQ862872–PQ862880 (9 isolates)	GenBank Accession Numbers	Country	Host	*16S rRNA* Nucleotide Positions													
						976	997	1095	1099	1106	1124	1134	1223	1225	1226	1228	1245	1277	1402
			PQ862877 **	TR	Goat	G	A	G	T	C	C	T	T	T	C	C	G	C	C
			OP828918	TR	W.Buf	*	*	*	*	*	*	*	*	*	*	*	*	*	*
			KJ782386	CN	Sheep	*	*	*	*	*	*	*	*	*	*	*	*	*	*
			KU321299	CN	Goat	*	*	*	*	*	*	*	*	*	*	*	*	*	*
			MT881655	TR	Sheep	*	*	*	*	*	*	*	*	*	*	*	*	*	*
		*A. phagocytophilum*-like 2 PQ862871 (1 isolate)	PQ862871 **	TR	Goat	*	G	*	*	T	*	C	C	C	T	*	A	T	*
			MG668805	CN	Tick	*	G	*	*	T	*	C	C	C	T	*	A	T	*
			MN194011	CN	Cattle	*	G	*	*	T	*	C	C	C	T	*	A	T	*
			KX702980	TN	Sheep	*	G	*	*	T	*	C	C	C	T	*	A	T	*
			KJ782381	CN	Sheep	*	G	*	*	T	*	C	C	C	T	*	A	T	*

*: unchanged nucleotide; **: This study isolates.

## Data Availability

The datasets presented in this study can be found in online repositories. The names of the repository/repositories and accession number(s) can be found below: https://www.ncbi.nlm.nih.gov/genbank/ accessed on 10 January 2025, PQ878024–PQ878071; PQ862871–PQ862880.
